# Functional adrenal insufficiency among tuberculosis-human immunodeficiency virus co-infected patients: a cross-sectional study in Uganda

**DOI:** 10.1186/s13104-020-05064-8

**Published:** 2020-04-19

**Authors:** Agnes Bwanika Naggirinya, Andrew Mujugira, David B. Meya, Irene Andia Biraro, Ezekiel Mupere, William Worodria, Yukari C. Manabe

**Affiliations:** 1 Department of Internal Medicine, School of Medicine, College of Health Sciences, Makerere University, Kampala, Uganda; 2 Infectious Diseases Institute, College of Health Sciences, Makerere University, Kampala, Uganda; 3 Department of Epidemiology and Biostatistics, School of Public Health, College of Health Sciences, Makerere University, Kampala, Uganda; 4 Department of Medicine, Division of Infectious Diseases, Johns Hopkins University, Baltimore, MD USA; 5 Department of Pediatrics, School of Medicine, College of Health Sciences, Makerere University, Kampala, Uganda; 6 Immodulation and Vaccines Programme, Medical Research Council/Uganda Virus Research Institute and London School of Hygiene & Tropical Medicine Uganda Research Unit, Entebbe, Uganda

**Keywords:** Adrenal, Insufficiency, HIV, TB, Africa

## Abstract

**Objective:**

Tuberculosis (TB) is the leading cause of adrenal insufficiency in resource-limited settings. The adrenal gland is the most commonly affected endocrine organ in TB infection. We assessed factors associated with functional adrenal insufficiency (FAI) among TB-HIV patients with and without drug-resistance in Uganda. Patients with drug-sensitive and drug-resistant TB were enrolled and examined for clinical signs and symptoms of FAI with an early morning serum cortisol level obtained. FAI was defined as early morning serum cortisol < 414 nmol//L. Associations with FAI were modeled using multivariable logistic regression.

**Results:**

We screened 311 TB patients and enrolled 272. Of these, 117 (43%) had drug-resistant TB. Median age was 32 years (IQR 18–66) and 66% were men. The proportion with FAI was 59.8%. Mean cortisol levels were lower in participants with drug-resistant than susceptible TB (317.4 versus 488.5 nmol/L; p < 0.001**)**. In multivariable analyses, drug-resistant TB (aOR 4.61; 95% CI 2.3–9.1; p **< **0.001), treatment duration > 1 month (aOR 2.86; 95% CI 1.4–5.5; p = 0.002) and abdominal pain (aOR 2.06; 95% CI 1.04–4.09; p = 0.038) were significantly associated with FAI. Early morning serum cortisol levels should be quantified in TB-HIV co-infected patients with drug-resistant TB.

## Introduction

Functional adrenal insufficiency (FAI), subnormal corticosteroid production during acute illness, results in high morbidity and mortality in critically ill patients [1, 2]. FAI is common among HIV-positive patients, with incidence rates up to 75% [3]. *Mycobacterium tuberculosis* and cytomegalovirus (CMV) infection [4] are the commonest etiologies of FAI, although it has also been described with *Cryptococcus neoformans*, *Toxoplasma gondii*, *Pneumocystis jiroveci*, non-Hodgkin’s lymphoma and Kaposi’s sarcoma [5, 6]. Adrenal tuberculosis (TB) results in destruction of the adrenal cortex leading to adrenal insufficiency if left untreated. Adrenal dysfunction may result from infectious infiltration of adrenal gland, inhibition of steroid synthesis by antifungal drugs used to treat opportunistic infections, stimulation of cytochrome P450 enzyme activity by rifampicin resulting in increased metabolism of cortisol, and cytokine abnormalities associated with HIV infection [7–9]. Adrenal TB leads to adrenal insufficiency through direct glandular involvement, extra-adrenal infection or as a result of anti-TB therapy [10]. Patients with drug-resistant TB (DR-TB) have significantly longer duration of disease than those with drug-sensitive (DS) TB [11], remain sputum positive for longer and have higher bacteriologic loads which increases risk of disseminated TB [11]. Subclinical FAI occurs in 23% of persons with pulmonary TB (PTB) infection [12], and is prevalent among patients with both DS and DR TB [11]. TB and HIV co-infection may compromise adrenocortical function and produce significant adrenocortical insufficiency [13].

The World Health Organization (WHO) estimates that 190,000 people died of multi-drug resistant TB (MDR-TB) in 2017 [14]. Globally, MDR-TB occurred in 3.5% of new and 18% of previously treated cases in 2017 [14]. In Uganda, a high TB-HIV burden country, prevalence of MDR-TB was 1.6% among newly diagnosed patients and 12.0% among previously treated patients [15]. Among severely ill HIV-positive adults in Uganda, FAI occurred in 19%, and those receiving rifampicin had 11 times higher odds of FAI [16]. A retrospective analysis of 13,762 patients (13,492 autopsies and 270 adrenalectomies) in Hong Kong found that active TB was present in 871 (6.5%) of 13,492 autopsies performed [17].

The diagnosis of FAI is often missed or delayed [18]. Delayed diagnosis often causes adrenal crisis, and increased morbidity and mortality [19]. Adrenocortical dysfunction is a known comorbidity of MDR-TB [20]. One study found low prevalence of FAI among HIV-negative MDR-TB patients in Mexico, a low TB burden country [20]. We assessed factors associated with FAI among TB patients in Uganda, a high TB burden country. We hypothesized that those with drug-resistant disease may be at higher risk of FAI.

## Main text

### Materials and methods

#### Study design and setting

From September 2015 to February 2016, we conducted a cross-sectional study at Mulago National TB Treatment Centre, in Kampala, Uganda, which has 60 and 40 beds for patients with DS-TB and DR-TB, respectively. Sample size estimation was guided by the difference between 2 proportions formula, assuming a 5% standard error, previous prevalence of FAI of 46% in DR-TB and 36% in DS-TB [16], giving a size of 191 for each group.

#### Participants

We consecutively sampled in-patients and used convenience sampling for out-patients diagnosed with TB. Participants were referred by clinical staff. A research assistant stationed at the TB Center from Monday to Saturday provided information about the study and obtained written informed consent prior to enrollment. We verified drug resistance and HIV status by reviewing medical files. We excluded patients without Xpert^®^ MTB/RIF or drug susceptibility testing (DST) results, history of steroid use in the prior 5 days, fluconazole use, pregnancy, diabetes mellitus, extrapulmonary TB or inability to provide informed consent (refusal, language barrier or unconscious with no caregiver).

#### Variables

An interviewer-administered questionnaire and the participant treatment card were used to collect data on patient demographics, medical history, HIV treatment status, past and current TB treatment, drug-susceptibility test results, and primary or secondary drug-resistance. Physical examination was performed to assess hyperpigmentation (buccal mucosa, palms, scarred skin), and postural hypotension to assess for signs of adrenocortical failure.

#### Data collection and outcome measures

Functional Adrenal Insufficiency, the primary outcome, was defined as early morning serum cortisol ≤ 414 nmol/L [2]. Blood was drawn between 0700 and 0930 h for serum cortisol, potassium, sodium, calcium, and complete blood count. Total serum cortisol was measured using the Cortisol ELISA Test (Diagnostic Automation Inc., CA, USA) with detection range 1–100 ng/ml (0.32–31.45 nmol/L). Diagnosis of rifampicin resistant-TB was performed using the Xpert^®^ MTB/RIF assay (Cepheid, Sunnyvale, CA, USA). Drug-susceptibility testing for multidrug resistance was performed using the BACTEC MGIT 960 system (Becton–Dickinson Microbiology System, Sparks, NV, USA) and Löwenstein-Jensen (L-J) media.

#### Statistical analysis

Data were entered into Epi-Data version 3.1 and exported to Stata version 13.0 (StataCorp, College Station, TX, USA) for analysis. A Student t-test was used to assess differences in serum cortisol between DR-TB and DS-TB participants. Associations with FAI were modeled using binary logistic regression. Variables significant (p < 0.2) in univariate analyses were included in the multivariate model. Two-sided p ≤ 0.05 were considered statistically significant.

### Results

#### Participant characteristics

Of the 311 participants (213 in-patients and 98 out-patients) screened, 272 were enrolled. Of these, 155 (57%) had drug-susceptible TB (DS-TB) and 117 had DR-TB (Additional file 1: Figure S1). A total of 154 (57%) were co-infected with HIV of whom 85 (57%) had DS-TB and 69 had DR-TB. The median age was 32 years (interquartile range [IQR] 18–66) and 180 (66%) were men. Baseline characteristics were comparable between DR-TB and DS-TB participants (Table 1). A total of 20 participants (7.4%) were TB treatment naïve. The median duration of TB treatment was 4.6 months (IQR 1–6), and the proportion with primary and secondary DR-TB was similar (51% versus 49%, respectively). Compared with DR-TB patients, a larger proportion of DS-TB patients had clinical features of weight loss, including prominent zygoma (72.5% versus 54.7%; p = 0.002) and prominent supraclavicular fossa (82.3% versus 69.0%; p = 0.01) (Table 1).

**Table 1:** Participant characteristics

Demographic characteristics	DS-TB (n = 155) N (%)	DR-TB^a^ (n = 117) N (%)	p-value
Age (years)			0.783
≤ 30	82 (55.0)	62 (53.5)	
31–45	50 (33.6)	43 (37.1)	
≥ 46	18 (11.4)	11 (9.5)	
Sex			0.355
Male	103 (66.7)	72 (62.2)	
Female	52 (33.3)	45 (38.8)	
HIV status			0.404
Positive	85 (54.8)	69 (59.0)	
Negative	70 (45.2)	48 (41.0)	
HIV treatment			< 0.001
ART naïve	38 (47.5)	4 (6.1)	
TDF-based regimens	27 (33.8)	40 (60.6)	
Other regimens	15 (18.8)	22 (33.3)	
TB treatment history			< 0.001
Category 1	117 (94.4)	52 (70.3)	
Category 2	7 (5.6)	22 (29.7)	
Current TB treatment duration			< 0.001
< 1 month	102 (68)	32 (28)	
< 1 month	49 (32)	84 (72)	
History of abdominal pain			0.784
No	64 (41.8)	47 (41.9)	
Yes	89 (58.5)	70 (58.1)	
History of weight loss			< 0.001
No	13 (8.5)	30 (25.6)	
Yes	140 (91.5)	87 (74.4)	
Clinical characteristics			
Weight loss-prominent zygoma			0.002
No	42 (27.5)	53 (45.3)	
Yes	111 (72.5)	64 (54.7)	
Weight loss-supraclavicular fossa			0.010
No	27 (17.7)	36 (31.0)	
Yes	126 (82.3)	80 (69.0)	
Serum cortisol (nmol/L)			< 0.001
< 414	64 (42.1)	97 (82.9)	
> 414	88 (57.9)	20 (17.1)	
Sodium [Na] (mmol/L)			< 0.001
Low (< 135)	43 (27.7)	12 (10.3)	
Normal (≥ 135)	112 (72.3)	105 (89.7)	
Potassium [K] (mmol/L)			0.043
≤ 5.0	125 (80.7)	104 (89.7)	
> 5.0	30 (19.3)	12 (10.3)	
Haemoglobin (g/dL)			< 0.001
≤ 9	46 (29.7)	10 (8.6)	
> 9	109 (70.3)	106 (91.4)	

Category 1 TB treatment for new smear positive pulmonary TB (6 months of Isoniazid, rifampicin, and initial 2 months of ethambutol, pyrazinamide)

Category 2 TB treatment Sputum smear positive who have relapsed or who have treatment failure or who are receiving treatment after treatment interruption (8 months of isoniazid, rifampicin and ethambutol supplemented by streptomycin for initial 2 months, and pyrazinamide for initial 3 months)

^a^56 participants had primary DR-TB and 54 had secondary DR-TB. Data were missing for 7 participants

#### Functional adrenal insufficiency

**Fig. 1:**
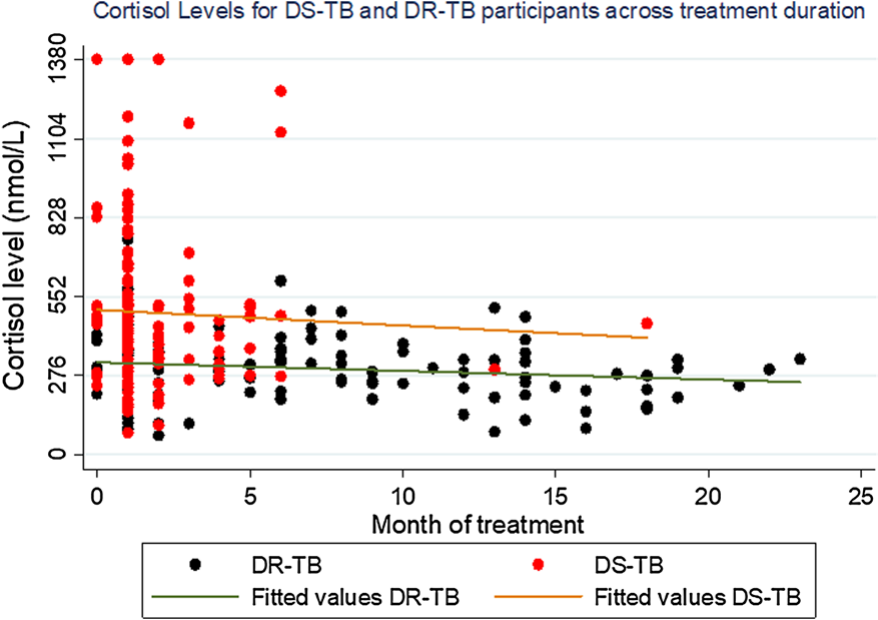
Cortisol Levels for DS-TB and DR-TB participants across treatment duration

The proportion with FAI was 59.8%. The median serum cortisol was 414.27 nmol/L (IQR 65.68–1380.00). DR-TB participants were more likely to have low basal morning [AM] serum cortisol (≤ 414 nmol/L) levels compared to DS-TB (82.9% versus 42.1%; p < 0.001). Similarly, mean cortisol levels were significantly lower in DR-TB participants than DS-TB (317.4 versus 488.5 nmol/L; p < 0.001). Cortisol levels remained lower among DR-TB participants irrespective of treatment duration (Fig. 1).

#### Associations with FAI

In multivariate analyses, participants with DR-TB had 5-times higher odds of FAI (adjusted odds ratio [aOR] 4.61; 95% confidence interval [CI] 2.3–9.1; p < 0.001) [Additional file 2: Table S1]. Participants with FAI had higher odds of abdominal pain (aOR 2.06; 95% CI 1.04–4.09; p = 0. 038), but lower odds of skin hyperpigmentation (darkened palms and buccal mucosa) (aOR 0.44; 95% CI 0.23–0.87; p =0.02). Being male was not associated with FAI (aOR 0.81; 95% CI 0.43–1.5; p = 0.53). Subgroup analyses revealed that DS-TB patients with FAI had higher odds of longer treatment duration and 70% lower odds of skin hyperpigmentation (Additional file 3: Table S2 and Additional file 4: Table S3).

## Discussion

In this cross-sectional study of 272 HIV-TB co-infected adults in Uganda, approximately two-thirds had FAI. Mean serum cortisol levels were significantly lower among DR-TB participants, who had five times higher odds of FAI compared with DS-TB patients. The odds of FAI were higher with DR-TB co-infection, history of abdominal pain and treatment duration > 1 month, but lower among men and participants with skin hyperpigmentation.

We found that a higher proportion of participants in our study had FAI than previously reported in India [11], Nigeria [12] and Uganda [16, 21], perhaps because of inclusion of persons with HIV co-infection which causes infectious adrenalitis [16]. By contrast, a study in Mexico among HIV-negative DR-TB patients, with equal numbers of primary and secondary multi-drug resistant TB cases, found a baseline prevalence of 4.2% using a serum cortisol cutoff of 500 nmol/L, and a prevalence of 8.3% when the cutoff was 550 nmol/L [20]. Delayed diagnosis and initiation of TB treatment in our setting may have led to hematogenous spread to the adrenal glands. Additionally, previous use of rifampicin, which enhances cortisol metabolism [16, 22], may account for the high occurrence of FAI observed. Our data suggest that clinical management of TB-HIV co-infected patients should include assessment of adrenal function because FAI increases morbidity and mortality [23].

DR-TB participants had lower mean cortisol levels and higher odds of FAI than DS-TB. Prevalence of DR-TB is higher in previously treated TB patients [24], and the lower cortisol levels we observed in DR-TB patients were likely due to delays in initiating TB treatment or to previous TB episodes increasing the likelihood of adrenal gland infiltration [25]. Additionally, standard TB treatment regimens include rifampicin which accelerates cortisol breakdown resulting in low cortisol levels; treatment duration > 1 month was associated with FAI among DS-TB patients in subgroup analyses [26]. These findings are in agreement with prior studies suggesting an association of adrenal insufficiency with rifampicin which is used for DS-TB treatment categories 1 and 2 [27]. We found that men had lower odds of adrenal insufficiency. Although TB is more prevalent in males, estrogen increases hepatic cortisol-binding globulin which lowers active free cortisol [18, 28]. Our finding agrees with prior work that showed that males have significantly higher free cortisol levels than females [29].

Infectious adrenalitis can be difficult to recognize clinically [30]. In low-income settings, where synthetic ACTH is not readily available for the gold-standard stimulation test, relying on symptoms and signs may result in incorrect or delayed diagnosis [18]. However, symptoms including weakness, fatigue, anorexia, nausea, vomiting, abdominal pain, myalgia, arthralgia, postural dizziness, craving for salt, headache, depression and memory impairment may be suggestive of FAI [16, 31, 32]. In our study, abdominal pain and treatment duration were associated with FAI, contrary to prior studies in which primary adrenal insufficiency was associated with hyperpigmentation of the buccal mucosa, palms and scarred skin [21, 30]. Darkening of the palms, persistent postural dizziness, fatigability, profound general body weakness, and syncope have been reported in a case of HIV-associated Addison’s disease without symptoms of PTB [33]. Skin hyperpigmentation is a specific sign of adrenal insufficiency, due to stimulation of melanocortin-1 receptors by the high levels of corticotrophin hormone resulting from lack of feedback from reduced cortisol levels [34].

In agreement with prior work [21], we found that history of abdominal pain was more likely among participants with FAI. Abdominal, flank and back pain is a common presentation in acute adrenal crisis mainly due to increased adrenal blood flow induced by increased secretion of ACTH [35]. Clinical manifestations of adrenal insufficiency result from deficiency of adrenocortical hormones secondary to adrenal cortex destruction; in subgroup analyses, DS-TB patients with FAI had 2-times higher odds of abdominal pain [34, 36]. Hypotension in adrenal insufficiency is caused by increases in arginine vasopressin, reduced aldosterone and sodium wasting [35]. Prior work in Uganda, found that 43% of patients with FAI had systolic hypotension and 19% had postural hypotension. Other work in the same setting found that 50% participants with adrenal insufficiency had systolic hypotension, and 29.2% had mucosal hyperpigmentation [21].

## Conclusions

FAI was common among TB-HIV co-infected adults as assessed by early morning cortisol levels. Presence of DR-TB, abdominal pain and longer treatment duration were associated with FAI. These factors may help clinicians in high TB-HIV burden and resource-limited settings to identify FAI and should be confirmed with early morning cortisol to guide clinical management.

## Limitations

A strength of our study is the inclusion of DR-TB and DS-TB participants, and the high prevalence of TB-HIV co-infection (50%). A limitation of this cross-sectional study is the inability to determine temporal relationships of TB and HIV infection in dually infected individuals thus limiting our analysis of clinical correlates of FAI. We were unable to perform sensitivity analyses to evaluate rifampicin-induced adrenal insufficiency due to the small number of TB treatment-naïve participants. Exclusion of patients without Xpert^®^ MTB/RIF or DST results or those unable to consent, probably removed sicker participants with FAI. We did not have access to abdominal CT scan to assess adrenal morphology. Finally, the gold-standard ACTH stimulation test was not available, and we may have misclassified FAI status in some participants.

## Supplementary information

**Additional file 1: Figure S1.** Study flow diagram. Study diagram describing the number of participants screened and enrolled and the reasons for screen-out. This to be inserted at end of line 127 on page 6.

**Additional file 2: Table S1.** Associations with Functional Adrenal Insufficiency. Factors associated with functional adrenal insufficiency. This is to be inserted under results section at end of line 147 on page 7.

**Additional file 3: Table S2.** Associations with functional adrenal insufficiency among DS-TB patients. Factors associated with FAI among drug-susceptible TB patients. This is to be inserted under results section at end of line 153 on page 7.

**Additional file 4: Table S3.** Associations with functional adrenal insufficiency among DR-TB patients. Factors associated with FAI among drug-resistant TB patients. This to be inserted at end of line 153 on page 7.

## Data Availability

All the data supporting the findings is submitted with the manuscript and in 2 additional supporting files.

## Abbreviations

ACTH: Adreno corticotropic hormone
CMV: Cytomegalovirus
CT: Computerized tomography
DR-TB: Drug-resistant tuberculosis
DST: Drug susceptibility test
DS-TB: Drug-sensitive tuberculosis
ELISA: Enzyme linked immuno sorbent assay
FAI: Functional adrenal insufficiency
HIV: Human immunodeficiency virus
IQR: Inter quartile range
TB: Tuberculosis
