# Supplementary figures and images for: Functional adrenal insufficiency among tuberculosis-human immunodeficiency virus co-infected patients: a cross-sectional study in Uganda
Source: BMC Res Notes. 2020 Apr 19;13:224. doi: 10.1186/s13104-020-05064-8 (PMC7169013; doi:10.1186/s13104-020-05064-8)

**Figure S1: Study Profile**

**
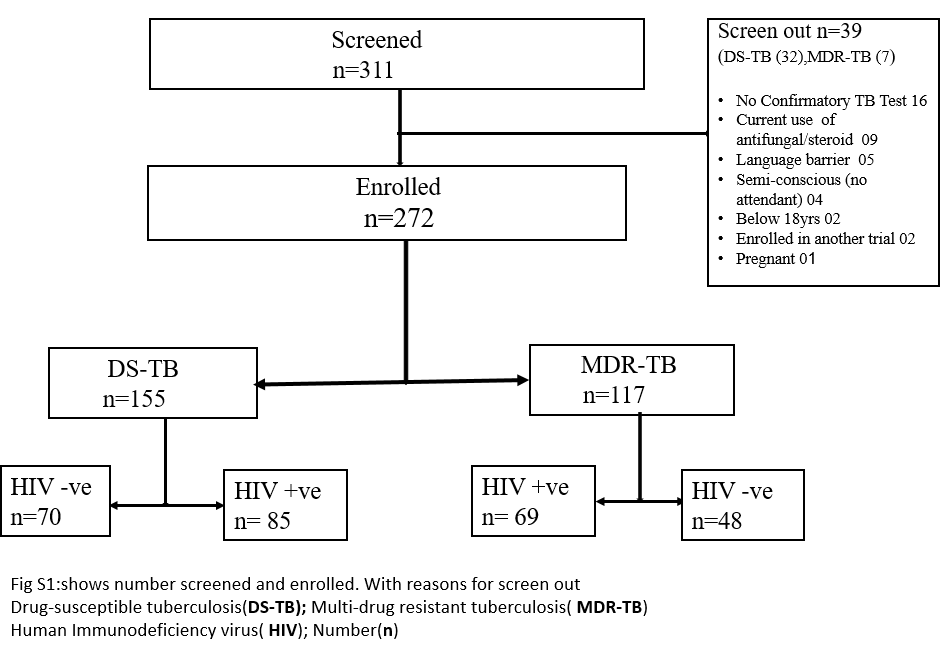
**

Supplement: Supplementary file 1 — Additional file 1: Figure S1. Study flow diagram. Study diagram describing the number of participants screened and enrolled and the reasons for screen-out. This to be inserted at end of line 127 on page 6. [file 13104_2020_5064_MOESM1_ESM.docx]
