# Supplementary material for: Functional adrenal insufficiency among tuberculosis-human immunodeficiency virus co-infected patients: a cross-sectional study in Uganda
Source: BMC Res Notes. 2020 Apr 19;13:224. doi: 10.1186/s13104-020-05064-8 (PMC7169013; doi:10.1186/s13104-020-05064-8)
Supplement: Supplementary file 2 — Additional file 2: Table S1. Associations with Functional Adrenal Insufficiency. Factors associated with functional adrenal insufficiency. This is to be inserted under results section at end of line 147 on page 7. [file 13104_2020_5064_MOESM2_ESM.docx]

| **Table S1: Associations with Functional Adrenal Insufficiency** | | |
| --- | --- | --- |
| **Clinical characteristics** | **Adjusted Odds Ratio (95% CI)** | **p-value** |
| **TB status** |  |  |
| DS-TB | Reference |  |
| DR-TB | 4.61 (2.3-9.1) | **<0.001** |
| **Gender** |  |  |
| Female | Reference |  |
| Male | 0.81 (0.43- 1.5) | 0.538 |
| **Current treatment duration** |  |  |
| <1 month | Reference |  |
| >1 month | 2.86 (1.4-5.5) | **0.002** |
| **Abdominal pain** |  |  |
| No | Reference |  |
| Yes | 2.06 (1.04-4.09) | **0.038** |
| **Weight loss** |  |  |
| No | Reference |  |
| Yes | 0.389 (0.134-1.12) | 0.081 |
| **Skin hyperpigmentation** |  |  |
| No | Reference |  |
| Yes | 0.44 (0.229-0.872) | **0.018** |
| **Laboratory characteristics** |  |  |
| Sodium (mmol/dL) |  |  |
| Normal (>135) | Reference |  |
| Low (<135) | 0.812 (0.384-1.717) | 0.586 |
| Potassium (mmol/dL) |  |  |
| >5.0 | Reference |  |
| ≤5.0 | 2.17 (0.966-4.88) | 0.061 |
| Hemoglobin (g/dl**)** |  |  |
| >9 | Reference |  |
| ≤9 | 1.34 (0.607-2.95) | 0.468 |
