# Supplementary material for: Functional adrenal insufficiency among tuberculosis-human immunodeficiency virus co-infected patients: a cross-sectional study in Uganda
Source: BMC Res Notes. 2020 Apr 19;13:224. doi: 10.1186/s13104-020-05064-8 (PMC7169013; doi:10.1186/s13104-020-05064-8)
Supplement: Supplementary file 3 — Additional file 3: Table S2. Associations with functional adrenal insufficiency among DS-TB patients. Factors associated with FAI among drug-susceptible TB patients. This is to be inserted under results section at end of line 153 on page 7. [file 13104_2020_5064_MOESM3_ESM.docx]

| **Table S2: Factors associated with functional adrenal insufficiency among DS-TB patients** | | |
| --- | --- | --- |
| **Clinical characteristics** | **Adjusted Odds Ratio (95% CI)** | **p-value** |
| Sex |  |  |
| Female | Reference |  |
| Male | 1.18 (0.52-2.64) | 0.684 |
| Current treatment duration |  |  |
| <1 month | Reference |  |
| >1 month | 3.24 (1.37-7.65) | **0.007** |
| Abdominal pain |  |  |
| No | Reference |  |
| Yes | 2.19 (0.93-5.14) | 0.069 |
| Weight loss |  |  |
| No | Reference |  |
| Yes | 0.29 (0.65-1.30) | 0.108 |
| Skin hyperpigmentation |  |  |
| No | Reference |  |
| Yes | 0.27 (0.11-0.650) | **0.003** |
| Laboratory characteristics |  |  |
| Sodium **(**mmol/dL) |  |  |
| Normal (>135) | Reference |  |
| Low (<135) | 0.82 (0.34-1.95) | 0.662 |
| Potassium **(**mmol/dL) |  |  |
| ≤5.0 | Reference |  |
| >5.0 | 3.29 (1.15-9.36) | **0.025** |
| Hemoglobin (g/dl**)** |  |  |
| ≤9 | Reference |  |
| >9 | 2.37 (0.96-5.86) | 0.061 |
