# Supplementary material for: Functional adrenal insufficiency among tuberculosis-human immunodeficiency virus co-infected patients: a cross-sectional study in Uganda
Source: BMC Res Notes. 2020 Apr 19;13:224. doi: 10.1186/s13104-020-05064-8 (PMC7169013; doi:10.1186/s13104-020-05064-8)
Supplement: Supplementary file 4 — Additional file 4: Table S3. Associations with functional adrenal insufficiency among DR-TB patients. Factors associated with FAI among drug-resistant TB patients. This to be inserted at end of line 153 on page 7. [file 13104_2020_5064_MOESM4_ESM.docx]

| **Table S3: Factors associated with adrenal insufficiency among DR-TB patients** | | |
| --- | --- | --- |
| **Clinical characteristics** | **Adjusted Odds Ratio (95% CI)** | **p-value** |
| Sex |  |  |
| Female | Reference |  |
| Male | 0.22 (0.049-1.04) | 0.057 |
| Current treatment duration |  |  |
| <1 month | Reference |  |
| >1 month | 2.63 (0.811-8.56) | 0.107 |
| Abdominal pain |  |  |
| No | Reference |  |
| Yes | 1.72 (0.431-6.936) | 0.440 |
| Weight loss |  |  |
| No | Reference |  |
| Yes | 0.36 (0.071-1.83) | 0.219 |
| Skin hyperpigmentation |  |  |
| No | Reference |  |
| Yes | 0.87 (0.233-3.29) | 0.847 |
| **Laboratory characteristics** |  |  |
| Sodium **(**mmol/dL) |  |  |
| Normal (>135) | Reference |  |
| Low (<135) | 2.56 (0.32-20.51) | 0.321 |
| Potassium **(**mmol/dL) |  |  |
| ≤5.0 | Reference |  |
| >5.0 | 1.14 (0.20-6.50) | 0.877 |
| Hemoglobin (g/dl**)** |  |  |
| >9 | Reference |  |
| ≤9 | 0.097 (0.89-204.47) | 0.060 |
